# HPV detection in first void urine and cervicovaginal samples: comparative study and analysis of associated factors in women from tunja, colombia

**DOI:** 10.1016/j.pmedr.2026.103393

**Published:** 2026-01-25

**Authors:** Lorenzo Hernando Salamanca Neita, Elizabeth Guío Mahecha, Mónica Gabriela Huertas Valero, Johana Marín Suárez, Juan Pablo Carvajal Rojas, Gloria Eugenia Camargo Villalba, Laura Ximena Ramírez López

**Affiliations:** a Carvajal Laboratorios IPS S.A.S 24th Street # 93-8 Tunja, Boyacá, Colombia; b Universidad de Boyacá Career 2 East, # 64-169 Tunja, Boyacá, Colombia

**Keywords:** Human papillomavirus, Detection, Urine, Cervicovaginal samples, Concordance, Sensitivity, Specificity

## Abstract

**Objective:**

To compare Human Papillomavirus (HPV) detection in first-void urine (FVU) and cervicovaginal samples and to analyze factors associated with cervicovaginal HPV infection among women in Tunja, Colombia.

**Methods:**

A cross-sectional study was conducted among 161 women aged 20–65 years residing in Tunja, Colombia. Samples and data were collected between September and November 2022 in a clinical laboratory. Cervicovaginal and FVU samples were obtained for HPV genotyping using the INNO-LiPA HPV Genotyping Extra II kit. Concordance, sensitivity, specificity, and associations with sociodemographic and gynecological variables were evaluated. Crude and adjusted odds ratios (OR) were estimated using logistic regression.

**Results:**

HPV prevalence was 29.19% in cervicovaginal samples and 32.91% in urine samples. Genotypes 52 and 68 were the most frequent in both sample types. Concordance between tests was weak (kappa = 0.39). Sensitivity and specificity of urine-based detection were 61.70% and 78.94%, respectively. In the multivariable model, only pregnancy history remained independently associated with cervicovaginal HPV infection.

**Conclusions:**

HPV infection prevalence was moderate, with high-risk genotypes detected in both sample types. The history of pregnancy was the only independent factor associated with cervicovaginal HPV infection. HPV detection in urine showed limited diagnostic performance, highlighting the need to optimize urine screening strategies.

## Highlights

• Urine and cervicovaginal samples showed similar human papillomavirus detection.
• High risk genotypes were common in urine and cervicovaginal samples.
• Urine testing showed limited agreement with cervicovaginal results.
• Sexual and reproductive factors were linked to cervicovaginal infection.
• Pregnancy history remained associated after multivariable adjustment.

## 1 Introduction

Human papillomavirus (HPV) is the most common sexually transmitted viral infection worldwide, with up to 80% of sexually active women acquiring it at least once (Doorbar et al., 2020). While most infections are transient, persistent infection may progress to cervical intraepithelial neoplasia and cervical cancer, a largely preventable malignancy when effective screening programs are implemented (Perkins et al., 2023). Cytology-based screening has traditionally been the cornerstone of prevention; however, its limited sensitivity for detecting high-grade lesions remains a challenge, particularly in low- and middle-income settings where coverage is suboptimal due to discomfort, logistical barriers, and limited access to trained personnel (Koliopoulos et al., 2017; Farajimakin, 2024). High-risk HPV DNA testing is now the preferred primary screening method because of its greater sensitivity, although specificity is lower due to detection of transient infections (World Health Organization, 2021; Kelly et al., 2022). Self-collection has emerged as an alternative strategy, as self-collected samples analyzed with validated molecular assays show comparable diagnostic accuracy to clinician-collected samples and improve screening participation (Yeh et al., 2019). First-void urine (FVU) is a promising non-invasive sample for HPV screening. Meta-analyses report good concordance with cervical samples, particularly when preservative-based collection devices are used, and higher acceptability in under-screened populations (Bober et al., 2021; Tatara et al., 2024). Therefore, this study compared HPV detection in FVU and cervicovaginal samples and analyzed factors associated with cervicovaginal HPV infection among women in Tunja, Colombia.

From a public health perspective, optimizing HPV screening strategies is essential to achieve the World Health Organization's goal of eliminating cervical cancer as a public health problem. Innovative, non-invasive approaches that increase participation among hard-to-reach populations can reduce screening inequities and improve early detection. In Latin America, where cervical cancer incidence remains high, strengthening preventive programs through accessible and acceptable testing methods is a priority for health systems. Therefore, this study compared HPV detection in FVU and cervicovaginal samples and analyzed factors associated with cervicovaginal HPV infection among women in Tunja, Colombia.

## 2 Methods

### 2.1 Study design and population

An observational cross-sectional analytical study was conducted in 161 women aged 20–65 years living in Tunja, Boyacá, Colombia. Sample size was calculated assuming a 10% loss, an HPV prevalence of 10.60% (Torrado et al., 2018), and a 95% confidence level. The study was approved by the Ethics and Bioethics Committee of Universidad de Boyacá (RECT-147/2022).

Participants were recruited at a private clinical laboratory in Tunja. Sexually active women were eligible, while those with a prior diagnosis of cervical cancer were excluded. After receiving study information, participants provided written informed consent and completed a structured questionnaire on sociodemographic and gynecological characteristics. HPV vaccination status was not assessed because most participants were adults before vaccine introduction in 2012–2013, and national coverage declined after 2014 (Henríquez-Mendoza, 2020). Therefore, vaccination was unlikely to influence HPV prevalence or genotype distribution. Samples and data were collected from September 13 to November 3, 2022.

Some invited women declined participation but were not recorded to preserve anonymity. Only participants who consented were documented. All interested women were screened using inclusion and exclusion criteria; those ineligible were excluded before sampling, and no personal identifiers were retained, in accordance with ethical anonymity principles.

### 2.2 Measures

Each participant received a Colli-Pee® FV-5000 device (Novosanis, Belgium) designed to collect 20 mL of first-void urine (FVU) through a standardized funnel mechanism (Pattyn et al., 2019). Women were instructed to self-collect first-stream urine and to avoid urination for at least one hour before collection.

Samples were delivered to the clinical laboratory within 30–90 min after home collection, according to routine institutional flow. Participants handed the sealed Colli-Pee® device to study staff. The device contains Universal Transport Medium (UTM), which stabilizes cellular material and preserves HPV DNA during short-term transport at room temperature. Upon receipt, urine samples were stored at −20 °C following the study protocol.

Cervicovaginal samples were collected during the same appointment by a gynecologist using a Cervex-Brush™ (Rovers Medical Devices). The brush was placed in PreservCyt™ solution (Hologic), a liquid-based cytology medium compatible with HPV DNA testing. Samples were stored at −80 °C until molecular processing.

Urine samples were centrifuged to concentrate exfoliated epithelial cells. The supernatant was discarded, and the pellet resuspended in molecular-grade water and stored at −80 °C. This standardized pre-analytical procedure was applied uniformly to urine and cervicovaginal samples according to kit instructions.

For DNA extraction, 200 μL of each sample (both urine pellet suspension and cervicovaginal sample medium) were processed using the Nextractor NX-48S system ((Genolution, Seoul, Korea), according to the manufacturer's instructions. Genetic material from urine and cervicovaginal samples was processed simultaneously using the INNO-LiPA HPV Genotyping Extra II kit (Fujirebio, Ghent, Belgium).

In the first phase, viral DNA was amplified by Polymerase Chain Reaction (PCR) using the INNO-LiPA HPV Genotyping Extra II AMP kit. This kit is designed to amplify a region of approximately 65 base pairs within the L1 region of the HPV genome using a consensus primer system. The obtained amplicons were stored at −80 °C for subsequent hybridization. In the second phase, reverse line hybridization (LiPA) was performed and genotype identification using the INNO-LiPA HPV Genotyping Extra II kit. Amplicons obtained in the first phase were hybridized with specific oligonucleotide probes for 32 HPV genotypes, immobilized in parallel lines on a nitrocellulose membrane. Subsequently, incubation with detection and chromogenic reagents was performed to visualize lines corresponding to genotypes present in each sample.

Results were interpreted as positive or negative for HPV presence. Positive results were classified into high-risk genotypes (HPV 16, 18, 31, 33, 35, 39, 45, 51, 52, 56, 58, 59, and 68), probable high-risk (HPV 26, 53, 66, 70, 73, and 82), and low or unknown risk (HPV 6, 11, 40, 42, 43, 44, 54, 61, 62, 67, 81, 83, and 84), according to reactive lines visualized on the membrane.

### 2.3 Statistical analysis

Sensitivity and specificity of HPV detection in FVU were calculated using cervicovaginal HPV results as the reference test. A 2 × 2 contingency table was constructed with paired results, where sensitivity corresponded to the proportion of cervicovaginal HPV-positive cases that were also positive in urine, and specificity to the proportion of cervicovaginal HPV-negative cases that were also negative in urine.

To identify circulating HPV genotypes in the studied population, participant results were organized according to sample type, and HPV presence was classified qualitatively (positive or negative). With qualitative results, the concordance of tests in cervicovaginal and urine samples was calculated using Cohen's kappa index with a 95% confidence interval (CI), considering Landis' classification (Bober et al., 2021) for interpreting indices: <0.00 minimal; >0.00–0.20 insignificant; 0.21–0.40 weak; >0.41–0.60 moderate; 0.61–0.80 substantial; 0.81–1.00 almost perfect.

Hypothesis testing was performed using a significance level of *p* < 0.05. Categorical variables were summarized using frequencies and proportions, and continuous variables using means and standard deviations. Crude associations between cervicovaginal HPV detection and sociodemographic, reproductive, and sexual behavior variables were evaluated through bivariate analyses, and crude odds ratios (OR) with 95% confidence intervals (CI) were calculated. Variables showing statistical significance in crude analysis or biological plausibility were included in a multivariable logistic regression model to control confounding. Adjusted odds ratios (aOR) and 95% CI were estimated from this model, which was used exclusively to assess associations between cervicovaginal HPV detection and epidemiologically relevant variables, not as a predictive model. Associations were not estimated for urine results because urine-based HPV detection served as the index test in the comparative analysis rather than as the clinical reference for infection status. Additionally, sensitivity and specificity values of viral detection using urine samples were determined. All statistical analyses were performed using IBM SPSS Statistics software, version 28.

## 3 Results

### 3.1 Sociodemographic characteristics of population

The study included 161 participants, all of whom had complete questionnaire information and valid cervicovaginal and urine samples for processing and analysis. With regard to eligibility and invalid results, only one woman initially enrolled had an invalid HPV molecular test result; she was contacted according to the ethical protocol and offered a repeat sample collection, but she declined and was therefore not included in the final analysis. No other invalid results or withdrawals from the study were recorded. (Table 1).

**Table 1:** Sociodemographic Characteristics of the Study Population. Tunja, Colombia, September–November 2022.

Sociodemographic Characteristics of the Study Population			
		N	%
Education Level	Primary	9	5.59
	Secondary	34	21.11
	Technical	31	19.25
	Technology	23	14.28
	University	33	20.49
	Postgraduate	31	19.25
Origin	Urban	145	90.06
	Rural	16	9.93
Marital Status	Single	73	45.34
	Common law marriage	40	24.84
	Married	33	20.49
	Separated	11	6.83
	Widowed	1	0.62
	Divorced	3	1.86
Occupation	Housewife	13	8.07
	Self-employed	17	10.55
	Unemployed	1	0.62
	Employed	106	65.83
	Student	24	14.90
Socioeconomic Status	One	38	23.60
	Two	58	36.02
	Three	54	33.54
	Four	7	4.34
	Five	4	2.48

The participants' ages ranged from 18 to 65 years, with an average age of 37.18 years and a standard deviation of 11.59. The age distribution, according to Resolution 3280 of 2018 (*Colombia. Ministerio de Salud y Protección Social. Resolución número 3280 de* 2018 (Resolution No. 3280 of 2018). Bogotá, 2018), which considers life stages, was as follows: youth (18 to 28 years), 48 women (29.81%); adulthood (29 to 59 years), 111 women (68.94%); and old age (60 years and older), 2 participants (1.24%).

In Colombia, socioeconomic status is classified into six ordinal categories (1 to 6) established by the national government for administrative and public service purposes. These categories reflect the average living conditions of residential areas based on structural characteristics, access to utilities, and urban conditions. Table 1 presents information on the sociodemographic characteristics of the population.

### 3.2 Gynecological history

Menarche occurred between the ages of 10 and 18, with an average age of 13.15 years. The age at first sexual intercourse ranged from 12 to 31 years, with a mean of 18 years. Forty-two women (26.09%) reported having no children, while the rest indicated having between 1 and 5 children, with one child being the most common response (30.43%). Over the past year, 70.80% of the participants (114 women) reported having had only one sexual partner. Regarding sexual practices, 28.57% (46 women) reported having oral sex, and 7.45% (12 women) reported having anal sex. Concerning clinical history, only one woman reported having a previous *Chlamydia trachomatis* infection; however, she did not test positive for HPV in any of the samples analyzed.

When asked about family planning methods, 16.14% (26 women) used barrier methods such as condoms, while 83.86% used other contraceptive methods, including intrauterine devices, oral contraception, monthly or quarterly injectable contraception, subdermal implants, transdermal patches, and emergency contraception. Additionally, 21.73% (35 women) reported having undergone tubal ligation.

Regarding Pap smear, 93.78% of the participants (151 women) had undergone this test at some point in their lives, and of these, 63.35% (102 women) had done so in the past year or currently. Moreover, 83.85% of the participants reported that their Pap smear results were normal.

### 3.3 HPV detection and genotype distribution

The overall prevalence of HPV was 29.19% (47 women) in cervicovaginal samples, while the prevalence of HPV in urine samples was 32.91% (53 women). Multiple-type HPV infections were identified in 4.34% of cervicovaginal samples and 12.42% of urine samples. Eighteen participants had positive results in cervicovaginal samples but negative in urine samples for HPV. Conversely, 24 women had positive results in urine samples but negative in cervicovaginal samples. Additionally, 29 participants showed positivity in both cervicovaginal and urine samples.

Genotypes 52 and 68 were the most prevalent in both the cervix and urine samples. Genotypes 16, 45, 51, 52, 54, 58, and 66 were most prevalent only in cervicovaginal samples, while genotypes 51, 52, 53, 59, and 61 predominated in urine samples. There was a notable presence of high-risk genotypes in both types of samples analyzed (Table 2a).

**Table 2:** Distribution of human papillomavirus (HPV) genotypes by oncogenic risk category in cervicovaginal and first-void urine samples among women in Tunja, Colombia, September–November 2022: (a) summary by risk category and (b) genotype-specific distribution by sample type.

a. **Summary by oncogenic risk category**					
**Risk category**		**Cervicovaginal n (%)**		**Urine n (%)**	
High risk		27 (57.44)		24 (45.28)	
Probable high risk		9 (19.14)		9 (16.98)	
High + probable high risk		1 (2.12)		5 (9.43)	
High + low/unknown risk		0 (0.00)		8 (15.09)	
High + probable high + low/unknown risk		0 (0.00)		1 (1.88)	
Low/unknown risk		10 (21.27)		6 (11.32)	
b. **Genotype-specific distribution by sample type**					
	**Genotype**	**Both positive**	**Only cervicovaginal**	**Only urine**	**Both negative**
**High risk**	HPV16	1	2	1	157
	HPV18	1	1	1	158
	HPV31	0	0	2	159
	HPV33	0	0	1	160
	HPV35	1	0	1	159
	HPV39	0	1	1	159
	HPV45	0	2	1	158
	HPV51	3	2	7	149
	HPV52	5	2	4	150
	HPV56	2	0	1	158
	HPV58	2	2	2	155
	HPV59	2	0	3	156
	HPV68	3	1	1	156
**Probable high risk**	HPV26	1	0	0	160
	HPV53	2	1	3	155
	HPV66	2	2	2	155
	HPV70	2	0	2	157
	HPV73	0	0	0	161
	HPV82	1	0	2	158
**Low / unknown risk**	HPV6	0	0	1	160
	HPV11	0	0	0	161
	HPV40	0	0	0	161
	HPV42	0	0	0	161
	HPV43	0	0	0	161
	HPV44	2	1	1	157
	HPV54	0	2	1	158
	HPV61	0	0	6	155
	HPV62	1	0	0	160
	HPV67	2	0	0	159
	HPV81	0	1	1	159
	HPV83	0	0	1	160
	HPV89	0	1	2	158

Among the 47 women who tested positive for HPV in cervicovaginal samples, 7 exhibited the coexistence of two different genotypes, while 40 women presented only one genotype in these samples. Additionally, in the 53 women who tested positive in urine samples, it was found that 2 harbored four different genotypes, 4 women showed three genotypes, 14 women presented two genotypes, and 33 participants harbored only one genotype in urine samples. (Table 2b).

### 3.4 Concordance and test characteristics

The comparison of molecular HPV detection between cervicovaginal and urine samples revealed a weak concordance, indicated by a kappa index of 0.39. For a more detailed evaluation of the characteristics of the urine test compared to the cervicovaginal test, the latter was considered the gold standard or reference test. (Table 3).

**Table 3:** Diagnostic performance of human papillomavirus (HPV) detection in first-void urine compared with cervicovaginal samples as reference among women in Tunja, Colombia, September–November 2022.

Performance Characteristics of HPV Test in Urine	
	Result (95% CI)
Prevalence	32.91
Sensitivity	61.70 (44.01, 79.39)
Specificity	78.94 (64.10, 93.78)
Positive Predictive Value	54.71 (36.59, 72.83)
Negative Predictive Value	83.33 (69,76, 96,90)
Positive Likelihood Ratio	2.92 (−3.21,9.05)
Negative Likelihood Ratio	0.48 (−2.04, 3.00)

### 3.5 Factors associated with HPV presence

In crude analysis, cervicovaginal HPV infection was significantly associated with age 18–37 years, early sexual debut at 15 years or younger, menarche before 13 years, and pregnancy history. In the multivariable logistic regression model including age, age at sexual debut, menarche, and pregnancy history. Only pregnancy history remained independently associated with cervicovaginal HPV infection, while the other variables did not retain statistical significance after adjustment. (Table 4).

**Table 4:** Crude and adjusted odds ratios for factors associated with human papillomavirus (HPV) detection in cervicovaginal samples among women in Tunja, Colombia, September–November 2022.⁎, ⁎⁎

Cervicovaginal Sample					
Characteristic		Positive n	Crude OR (95% CI)	Adjusted OR (95% CI)	*p*-value (adjusted)
Age (years)	18–37	33	2.69 (1.31, 5.70)	0.58 (0.26, 1.31)	0.19
Education level	Higher	28	1.37 (0.68, 2.76)	–	–
Origin	Urban	45	3.13 (0.76, 21.1)	–	–
Marital status	With partner	39	1.03 (0.42, 2.69)	–	–
Socioeconomic status	Low (1–2)	29	1.05 (0.52, 2.14)	–	–
Pregnancy history	Yes	29	0.36 (0.17, 0.78)	2.37 (1.01, 5.59)	0.04
Contraceptive method	Other	43	2.55 (0.87, 9.14)	–	–
Sexual debut (years)	≤15	12	3.18 (1.27, 8.04)	0.40 (0.15, 1.07)	0.06
Pap smear history⁎	Yes	42	0.38 (0.09, 1.51)	–	–
Menarche (years)	<13	23	2.06 (1.02, 4.17)	0.53 (0.25, 1.13)	0.09
Oral sex⁎⁎	Yes	15	1.25 (0.58, 2.62)	–	–
Anal sex⁎⁎	Yes	4	1.23 (0.30, 4.29)	–	–

The reference category for each variable is the first category presented in the table, corresponding to the upper row within each categorical grouping.

⁎ Pap smear (yes/no) was defined as having had a vaginal cytology test at least once in the last 3 years.

⁎⁎ Sexual practices, including oral and anal sex, were recorded as frequent behaviors in the last year.

## 4 Discussion

Human papillomavirus (HPV) is one of the most common sexually transmitted infections worldwide (Tapia-Vela and Campuzano-Zuluaga, 2021). Although most HPV infections are transient and asymptomatic, persistent infection with high-risk genotypes can lead to precancerous lesions and cervical cancer. In Colombia, HPV infection is not a mandatory notifiable disease, which limits accurate estimates of its incidence and prevalence across regions (*Colombia. Ministerio de Salud y Protección Social. Encuesta Nacional de Demografía y Salud. Tomo I (National Demographic and Health Survey. Volume I). Bogotá*, 2015). Nonetheless, HPV infection remains a major public health concern, as it is a necessary cause of cervical cancer, one of the malignancies contributing substantially to years of life lost (YLL) among women, particularly those of young and middle age (*JAMA Oncol.*, 2022). The persistence of cervical cancer as a public health problem in Colombia is closely linked to socioeconomic inequalities and barriers to effective screening in low- and middle-income settings (Muñoz and Bravo, 2013).

In crude analysis, HPV positivity was associated with younger age (18–37 years; OR = 2.69), early sexual debut (≤15 years; OR = 3.18), and menarche before 13 years (OR = 2.06), consistent with the higher prevalence of HPV infection in younger women due to greater cumulative sexual exposure (Burger et al., 2017; Baisley et al., 2020; Morales-Figueroa et al., 2023; Terrinoni et al., 2025). After adjustment for reproductive and sexual life-course variables, only pregnancy history remained independently associated with HPV infection (aOR = 2.37; *p* = 0.04), indicating confounding among age, sexual debut, and reproductive factors. Pregnancy and multiparity may contribute to HPV persistence through cervical microtrauma, hormonal changes, and immune modulation (Condrat et al., 2021; Tekalegn et al., 2022; Jensen et al., 2013).

In Colombia, national guidelines recommend cervical cancer screening for women aged 25–65 years using HPV testing combined with cytology every five years or cytology alone every three years (*Colombia. Ministerio de Salud y Protección Social. Guía de Práctica Clínica para la detección y manejo de lesiones precancerosas de cuello uterino (Clinical Practice Guideline for the detection and management of cervical precancerous lesions). Guía No. GPC* 2014–44. Bogotá, 2014), in alignment with the 2021 WHO guideline, which endorses HPV DNA testing as the preferred primary screening method and recognizes self-collected samples as a valid alternative to improve access and coverage (World Health Organization, 2021). This shift responds to the limited sensitivity of cytology for detecting precancerous lesions and the higher sensitivity of HPV DNA–based screening, although the latter has lower specificity due to the frequent detection of transient infections (Origoni et al., 2012). Together, these considerations highlight the need for more precise diagnostic strategies capable of distinguishing infections with a higher risk of progression, thereby optimizing screening effectiveness and minimizing unnecessary interventions.

Global meta-analyses estimate HPV prevalence in women with normal cytology at 10–16%, with higher rates in sub-Saharan Africa and Latin America and lower rates in Europe and East Asia, suggesting a comparatively higher prevalence among women from Tunja (Sabeena et al., 2017; Bruni et al., 2010). The lower concordance observed may be explained by methodological and population differences, as studies with optimized pre-analytical conditions and assays focused on high-risk genotypes report higher agreement (kappa 0.41–0.82) (Cho et al., 2021; Martinelli et al., 2023). In contrast, our inclusion of low and probable high-risk genotypes and the absence of cervical lesions among participants may have resulted in lower viral loads and reduced concordance between sample types.

FVU is a promising non-invasive alternative for HPV detection and can achieve diagnostic accuracy comparable to that of clinician-collected samples when using standardized, preservative-based collection protocols (Arbyn et al., 2014). In this study, the lower sensitivity (61.7%) and specificity (78.9%), as well as the reduced concordance compared to previous reports, are likely explained by multiple factors, particularly pre-analytical handling. The protocol used (centrifugation and resuspension in water without preservative buffer) may compromise DNA stability and reduce viral detection, especially in urine samples (Miazga et al., 2024). Other contributing factors include differences in diagnostic assays, as LiPA-based genotyping and PCR methods vary in analytical sensitivity and target regions (Arbyn et al., 2014), as well as population-level factors such as genotype distribution and sexual behavior patterns. Taken together, these elements underscore the need for methodological optimization before FVU-based HPV testing can be widely implemented (Davies et al., 2024).

Molecular HPV testing improves risk stratification by identifying high-risk genotypes, particularly HPV16 and HPV18, which are strongly associated with high-grade lesions and cervical cancer (Li et al., 2022). Variability in genotype distribution across populations highlights the importance of local characterization, as progression depends not only on persistent oncogenic infection but also on additional molecular and host-related factors (Sisodiya et al., 2025). In this context, our study provides relevant data on HPV prevalence and genotyping in women from Tunja, Colombia, while underscoring the need to improve urine-based detection methods given the low concordance and diagnostic performance observed.

This study has several limitations that should be considered when interpreting the findings. First, the cross-sectional design precludes the assessment of HPV persistence and temporal changes in genotype distribution. Second, there is the issue of urine sample pretreatment and preservation. While standardized protocols recommend buffer solutions to improve cellular and DNA stability, this study followed the instructions for the commercial kit, which include centrifugation, resuspension in molecular-grade water, and storage at −80 °C. While this ensured consistent sample handling, it may differ from other validated approaches and influence analytical performance.

Another limitation relates to the analytical sensitivity of urine samples, which may be lower than that of cervicovaginal samples due to reduced cell content and variability in FVU capture, potentially introducing variability in HPV DNA yield. Third, assay-related factors, including differences in DNA extraction efficiency, PCR amplification, and hybridization interpretation, can affect genotype detection and concordance between sampling methods. Furthermore, cervicovaginal HPV detection was used as the reference standard; therefore, the reported sensitivity and specificity reflect concordance between sample types rather than diagnostic accuracy for underlying cervical disease.

Finally, the study did not assess HPV vaccination status, coinfections, or behavioral factors that may affect HPV prevalence and genotype distribution. Further longitudinal studies using standardized urine preservation methods and incorporating additional molecular biomarkers (e.g., viral load or E6/E7 mRNA) are warranted to strengthen the diagnostic utility of urine HPV testing.

## 5 Conclusions

The prevalence of HPV infection among women in Tunja was lower than that reported in national and international studies, with high-risk genotypes detected in both cervicovaginal and urine samples. Although urine-based HPV detection showed lower diagnostic performance and agreement in this study, these results should be interpreted in light of the specific pre-analytical and analytical procedures applied. Overall, the findings highlight the importance of considering reproductive life-course factors when interpreting HPV-related associations and when designing cervical cancer screening and sexual health education strategies.

## CRediT authorship contribution statement

**Lorenzo Hernando Salamanca Neita:** Writing – review & editing, Writing – original draft, Visualization, Validation, Supervision, Software, Resources, Project administration, Methodology, Investigation, Funding acquisition, Formal analysis, Data curation, Conceptualization. **Elizabeth Guío Mahecha:** Writing – review & editing, Writing – original draft, Visualization, Supervision, Resources, Project administration, Methodology, Investigation, Funding acquisition, Data curation, Conceptualization. **Mónica Gabriela Huertas Valero:** Writing – review & editing, Writing – original draft, Visualization, Supervision, Software, Methodology, Investigation, Conceptualization. **Johana Marín Suárez:** Writing – review & editing, Writing – original draft, Visualization, Validation, Software, Methodology, Investigation, Formal analysis, Data curation, Conceptualization. **Juan Pablo Carvajal Rojas:** Writing – review & editing, Writing – original draft, Visualization, Supervision, Resources, Project administration, Methodology, Investigation, Funding acquisition, Conceptualization. **Gloria Eugenia Camargo Villalba:** Writing – review & editing, Writing – original draft, Visualization, Supervision, Resources, Project administration, Methodology, Investigation, Funding acquisition, Conceptualization. **Laura Ximena Ramírez López:** Writing – review & editing, Writing – original draft, Visualization, Validation, Software, Project administration, Methodology, Investigation, Formal analysis, Data curation, Conceptualization.

## Declaration of competing interest

The authors declare that they have no known competing financial interests or personal relationships that could have appeared to influence the work reported in this paper.

## Data Availability

The data that has been used is confidential.

## References

[bb0005] Arbyn M., Verdoodt F., Snijders P.J., Verhoef V.M., Suonio E., Dillner L., Minozzi S., Bellisario C., Banzi R., Zhao F.H., Hillemanns P., Anttila A. (2014). Accuracy of human papillomavirus testing on self-collected versus clinician-collected samples: a meta-analysis. Lancet Oncol..

[bb0010] Baisley K.J., Andreasen A., Irani J., Nnko S., Changalucha J., Crucitti T. (2020). HPV prevalence around the time of sexual debut in adolescent girls in Tanzania. Sex. Transm. Infect..

[bb0015] Bober P., Firment P., Sabo J. (2021). Diagnostic test accuracy of first-void urine human papillomaviruses for presence cervical HPV in women: systematic review and Meta-analysis. Int. J. Environ. Res. Public Health.

[bb0020] Bruni L., Diaz M., Castellsagué X., Ferrer E., Bosch F.X., de Sanjosé S. (2010). Cervical human papillomavirus prevalence in 5 continents: meta-analysis of 1 million women with normal cytological findings. J. Infect. Dis..

[bb0025] Burger E.A., Kim J.J., Sy S., Castle P.E. (2017). Age of acquiring causal human papillomavirus (HPV) infections: leveraging simulation models to explore the natural history of HPV-induced cervical Cancer. Clin. Infect. Dis..

[bb0030] Cho H.W., Hong J.H., Min K.J., Ouh Y.T., Seong S.J., Moon J.H. (2021). Diagnostic accuracy of high-risk human papillomavirus assays on self-collected vaginal and urine samples for cervical cancer screening in referral population. Cancer Res. Treat..

[bb0035] (2015). Colombia. Ministerio de Salud y Protección Social. Encuesta Nacional de Demografía y Salud. Tomo I (National Demographic and Health Survey. Volume I). Bogotá.

[bb0040] (2014). Colombia. Ministerio de Salud y Protección Social. Guía de Práctica Clínica para la detección y manejo de lesiones precancerosas de cuello uterino (Clinical Practice Guideline for the detection and management of cervical precancerous lesions). Guía No. GPC 2014–44. Bogotá.

[bb0045] (2018). Colombia. Ministerio de Salud y Protección Social. Resolución número 3280 de 2018 (Resolution No. 3280 of 2018). Bogotá.

[bb0050] Condrat C.E., Filip L., Gherghe M., Cretoiu D., Suciu N. (2021). Maternal HPV infection: effects on pregnancy outcome. Viruses.

[bb0055] Davies J.C., Sargent A., Pinggera E., Carter S., Gilham C., Sasieni P., Crosbie E.J. (2024). Urine high-risk human papillomavirus testing as an alternative to routine cervical screening: a comparative diagnostic accuracy study of two urine collection devices using a randomised study design trial. BJOG.

[bb0060] Doorbar J., Egawa N., Griffin H., Kranjec C., Murakami I. (2020). Human papillomavirus molecular biology and disease association. Rev. Med. Virol..

[bb0065] Farajimakin O. (2024). Barriers to cervical Cancer screening: a systematic review. Cureus.

[bb0070] Henríquez-Mendoza G.M. (2020). El “evento de El Carmen de Bolívar” en la vacunación contra VPH en Colombia. ¿Causa o desenlace? (The “El Carmen de Bolívar” event in HPV vaccination in Colombia: cause or outcome?). Rev Salud Pública..

[bb0075] (2022). Global burden of disease 2019 Cancer collaboration. Cancer incidence, mortality, years of life lost, years lived with disability, and disability-adjusted life years for 29 Cancer groups from 2010 to 2019: a systematic analysis for the global burden of disease study 2019. JAMA Oncol..

[bb0080] Jensen K.E., Schmiedel S., Norrild B., Frederiksen K., Iftner T., Kjaer S.K. (2013). Parity as a cofactor for high-grade cervical disease among women with persistent human papillomavirus infection: a 13-year follow-up. Br. J. Cancer.

[bb0085] Kelly H., Jaafar I., Chung M., Michelow P., Greene S., Strickler H., Xie X., Schiffman M., Broutet N., Mayaud P., Dalal S., Arbyn M., de Sanjosé S. (2022). Diagnostic accuracy of cervical cancer screening strategies for high-grade cervical intraepithelial neoplasia (CIN2+/CIN3+) among women living with HIV: a systematic review and meta-analysis. EClinicalMedicine.

[bb0090] Koliopoulos G., Nyaga V.N., Santesso N., Bryant A., Martin-Hirsch P.P., Mustafa R.A., Schünemann H., Paraskevaidis E., Arbyn M. (2017). Cytology versus HPV testing for cervical cancer screening in the general population. Cochrane Database Syst. Rev..

[bb0095] Li X., Rao X., Wei M.J., Lu W.G., Xie X., Wang X.Y. (2022). Extended HPV genotyping for risk assessment of cervical intraepithelial neoplasia grade 2/3 or worse in a cohort study. J. Natl. Compr. Cancer Netw..

[bb0100] Martinelli M., Giubbi C., Di Meo M.L., Perdoni F., Musumeci R., Leone B.E. (2023). Accuracy of human papillomavirus (HPV) testing on urine and vaginal self-samples compared to clinician-collected cervical sample in women referred to colposcopy. Viruses.

[bb0105] Miazga W., Tatara T., Wnuk K., Gujski M., Pinkas J., Religioni U. (2024). The impact of urine-sample HPV testing on the effectiveness of screening for cervical Cancer: an umbrella review. Cancers (Basel).

[bb0110] Morales-Figueroa G.G., Bravo-Parra M., Olivas-Matas K.M., Esparza-Romero J., Valenzuela-Zamorano M., Olivas-López O.M., Quihui-Cota L. (2023). Associated factors with human papillomavirus (HPV) infection in adult women from Northwest Mexico. Biotecnia.

[bb0115] Muñoz N., Bravo L.E. (2013). Epidemiology of cervical cancer in Colombia. Colomb Med [Internet]..

[bb0120] Origoni M., Cristoforoni P., Costa S., Mariani L., Scirpa P., Lorincz A., Sideri M. (2012). HPV-DNA testing for cervical cancer precursors: from evidence to clinical practice. Ecancermedicalscience.

[bb0125] Pattyn J., Van Keer S., Biesmans S., Ieven M., Vanderborght C., Beyers K., Vankerckhoven V., Bruyndonckx R., Van Damme P., Vorsters A. (2019). Human papillomavirus detection in urine: effect of a first-void urine collection device and timing of collection. J. Virol. Methods.

[bb0130] Perkins R.B., Wentzensen N., Guido R.S., Schiffman M. (2023). Cervical Cancer screening: a review. JAMA.

[bb0135] Sabeena S., Bhat P.V., Kamath V., Bhat ShK, Nair S., n R, Chandrabharani K, Arunkumar G. (2017). Community-based prevalence of genital human papilloma virus (HPV) infection: a systematic review and Meta-analysis. Asian Pac. J. Cancer Prev..

[bb0140] Sisodiya S., Singh P., Joshi T., Aftab M., Firdausi N., Khan A., Mishra N., Jamil Khan N., Tanwar P., Gupta V., Hussain S. (2025). Human papillomavirus-mediated cervical cancer: epigenetic interplay and clinical implications. Front. Microbiol..

[bb0145] Tapia-Vela L.J., Campuzano-Zuluaga G. (2021). Laboratorio molecular en virus del papiloma humano (VPH) y cáncer (Molecular laboratory in human papillomavirus (HPV) and cancer). Medicina y. Laboratorio.

[bb0150] Tatara T., Wnuk K., Gujski M., Pinkas J., Religioni U. (2024). The impact of urine-sample HPV testing on the effectiveness of screening for cervical Cancer: an umbrella review. Cancers (Basel).

[bb0155] Tekalegn Y., Sahiledengle B., Woldeyohannes D., Atlaw D., Degno S., Desta F., Bekele K., Aseffa T., Gezahegn H., Kene C. (2022). High parity is associated with increased risk of cervical cancer: systematic review and meta-analysis of case-control studies. Womens Health (Lond)..

[bb0160] Terrinoni M., Golia D’Augè T., Mascellino G., Adinolfi F. (2025). Human papillomavirus across the reproductive lifespan: an integrative review of fertility, pregnancy outcomes, and fertility-sparing management. Medicina (Kaunas).

[bb0165] Torrado L., Rincón-Orozco B., Martínez-Vega R. (2018). Genotipificación del virus de Papiloma Humano en mujeres de la comuna norte de Bucaramanga (human papillomavirus genotyping in women from the northern district of Bucaramanga). Salud UIS..

[bb0170] World Health Organization. WHO Guideline for Screening and Treatment of Cervical Pre-cancer Lesions for Cervical cancer Prevention. Geneva: WHO; 2021. Available from: https://www.who.int/publications/i/item/9789240030824. 34314129

[bb0175] Yeh P.T., Kennedy C.E., de Vuyst H., Narasimhan M. (2019). Self-sampling for human papillomavirus (HPV) testing: a systematic review and meta-analysis. BMJ Glob. Health.

